# Computational identification of natural senotherapeutic compounds that mimic dasatinib based on gene expression data

**DOI:** 10.1038/s41598-024-55870-4

**Published:** 2024-03-15

**Authors:** Franziska Meiners, Burkhard Hinz, Lars Boeckmann, Riccardo Secci, Salem Sueto, Lars Kuepfer, Georg Fuellen, Israel Barrantes

**Affiliations:** 1 Institute for Biostatistics and Informatics in Medicine and Ageing Research, Rostock University Medical Center, Rostock, Germany; 2 Institute of Pharmacology and Toxicology, Rostock University Medical Center, Rostock, Germany; 3 Clinic and Policlinic for Dermatology and Venerology, University Medical Center Rostock, Strempelstr. 13, 18057 Rostock, Germany; 4 Institute for Systems Medicine with Focus on Organ Interaction, University Hospital RWTH Aachen, Aachen, Germany

**Keywords:** Computational biology and bioinformatics, Translational research, Drug discovery

## Abstract

The major risk factor for chronic disease is chronological age, and age-related chronic diseases account for the majority of deaths worldwide. Targeting senescent cells that accumulate in disease-related tissues presents a strategy to reduce disease burden and to increase healthspan. The senolytic combination of the tyrosine-kinase inhibitor dasatinib and the flavonol quercetin is frequently used in clinical trials aiming to eliminate senescent cells. Here, our goal was to computationally identify natural senotherapeutic repurposing candidates that may substitute dasatinib based on their similarity in gene expression effects. The natural senolytic piperlongumine (a compound found in *long pepper*), and the natural senomorphics parthenolide, phloretin and curcumin (found in various edible plants) were identified as potential substitutes of dasatinib. The gene expression changes underlying the repositioning highlight apoptosis-related genes and pathways. The four compounds, and in particular the top-runner piperlongumine, may be combined with quercetin to obtain natural formulas emulating the dasatinib + quercetin formula.

## Introduction

Cellular senescence was investigated by Hayflick and Moorhead as early as 1961. They found that cultured human fibroblasts could only undergo a certain number of replications until a state of replicative arrest was entered, now known as replicative senescence^1–3^. Non-replicative cellular senescence can be triggered by various factors including DNA damage, sustained inflammation, radiation, UVB light, DNA damaging chemotherapeutics, oncogene-activation, or tumor suppressor loss^4^. Senescent cells feature cell cycle arrest, but they do not undergo apoptosis and instead remain metabolically active, usually displaying the so-called senescence-associated secretory phenotype (SASP), the secretion of a diverse, often deleterious collection of pro-inflammatory cytokines, chemokines, proteases and lipids, leading to inflammation and tissue damage^5^, contributing to what is termed “inflammaging”, a chronic inflammation that is a common attribute in aged tissues^5–7^. Additionally, signaling pathways are activated that sustain their resistance to apoptosis and, in contrast to proliferating cells, senescent cells are believed to need these pathways, senescent cell anti-apoptotic pathways (SCAPs), in order to stay alive^8^. Resistance to apoptosis is a hallmark of senescent cells, primarily facilitated through upregulation of BCL2 family proteins; resistance to oxidative stress is another factor^4,9^.

Still, the accumulation of senescent cells can be subject to “senotherapeutic” intervention: by direct killing (senolysis), by modification of the SASP (senomorphics) or by slowing down the process by which cells become senescent (gerostatics). As a consequence, a lot of effort has been invested into finding drugs (termed senolytics) that kill senescent cells, e.g., by inhibiting anti-apoptotic pathways. By disabling these pro-survival pathways, they enable the selective elimination of senescent cells via the induction of apoptosis^10^. Target genes of senolytics include ephrins, BCL2- family proteins such as BCL2L1, PI3KCD and AKT, the transcription factor TP53, or the chaperone protein HSP90^8,11^. Unlike senolytics, senomorphics are drugs that can suppress SASP factors or hinder stressed cells from becoming senescent, e.g. by activation of the NRF2 or FOXO pathways, by decreasing/inhibiting NF-κB or mTOR activity, or by inhibiting the JAK-pathway^12–14^. We use the term “senotherapeutic” to cover senolytics and senomorphics.

Two known senolytics are dasatinib and quercetin, often studied in combination. Dasatinib is a drug developed for the treatment of leukemias, and it exerts its antitumoral activity by inhibition of SCR/ABL1 kinases, and by inhibiting the BCL-ABL1 fusion protein, the driving oncogene of chronic myeloid leukemia^15,16^. Quercetin is a polyphenol (flavonol) known as a potent natural compound, found in many fruit and vegetables^17,18^. The joint senolytic activity of the two compounds was discovered through a hypothesis-driven approach^5,19^. Dasatinib can act as a senolytic by interfering with ephrin-dependent suppression of apoptosis and inhibition of tyrosine kinases; quercetin can act as a senolytic by inhibiting PI3K/AKT, BCL2/BCL2L1, and TP53/P21/serpine SCAPs^5,19^.

Senolytics are cell-type specific (see Table 1); i.e. quercetin is effective in killing senescent HUVECs, and dasatinib kills senescent human preadipocytes^8^. Combining dasatinib and quercetin (D + Q) successfully reduced viability in both cell types^8^. Further, D + Q reduced abundance of senescent primary mouse embryonic fibroblasts and senescent bone marrow derived mesenchymal stem cells^8^, and induced apoptosis in senescent (fibrotic) alveolar epithelial type II cells, as was shown in an ex vivo model of lung fibrosis^20^. In murine models, D + Q prevented uterine age-related dysfunction and fibrosis, reduced intestinal senescence and inflammation and modulated the gut microbiome in aged mice, reduced senescent cell load in the context of age-related hepatic steatosis, protected retinal ganglion cell loss by early removal of senescent cells as well as improved metabolic function and reduced adipose tissue inflammation in old mice^21–25^. Long-term treatment by D + Q reduced the number of senescent cells and ameliorated age-dependent intervertebral disk-degeneration in mice, along with downregulation of circulating proinflammatory factors and an increase in physical strength^26^. In humans, D + Q showed improved disease-related outcomes e.g. leading to reduced adipose tissue senescent cell burden in individuals with diabetic kidney disease and it improved physical strength and function in patients with idiopathic pulmonary fibrosis^27,28^. However, as with other antitumor drugs, adverse events of dasatinib are frequent, such as respiratory events, skin irritation, myelosuppression, fluid retention events or diarrhea^27^. Hence, finding non-toxic analogs of dasatinib, especially from natural sources, possibly for combination with quercetin, would be of high value.

**Table 1:** The known senolytic compounds Dasatinib (D), Quercetin (Q), the combination of both (D + Q), and Piperlongumine, and the targeted senescent cell types.

Compound	Targeted senescent cell types	References
Piperlongumine	Human WI fibroblasts	^29^
Dasatinib (D)	Human and mouse preadipocytes	^8^
Quercetin (Q)	HUVECs	^8^
D + Q	Same cells as by D or Q Primary mouse embryonic fibroblasts Bone marrow-derived mesenchymal stem cell Alveolar epithelial type II cells senescent cells in skin ulcer samples	^8,20,30–32^

In this regard, traditional drug discovery is a time-consuming, costly and labor-intense process with high failure rates^33^. Computational methods to identify existing drugs for a new purpose (known as drug repositioning, or repurposing) offer an alternative to de novo drug discovery, as it imposes fewer risks, resources and economic effort^34–36^. A common repositioning approach is built on the hypothesis that if two drugs induce similar gene expression profiles and thus may be assumed to have similar modes of action, both could be considered to treat the same condition^34^. Transcriptomic gene expression profiles capture some of the dynamics of the cellular response to a drug intervention and measure the transcriptional activity of hundreds or thousands of genes simultaneously, and therefore help understanding how genes act under the same or similar circumstances^34^. Two key resources for drug repositioning are the Connectivity Map and the Library of Integrated Network-Based Cellular Signatures (LINCS) projects. The Connectivity Map is a database where genes, drugs and cell lines are connected by common gene expression signatures^37^. LINCS is a program funded by the National Institutes of Health to generate an extensive reference database of cell- based perturbation- response signatures based on different model systems including cell lines, differentiated cells and embryonic stem cells^38^. LINCS is an expanded version of the Connectivity Map and comprises over a million gene expression profiles of chemically perturbed human cell lines that can be used to discover mechanisms of action of small molecules, based on a compacted representation of the transcriptome^37,39^.

The repositioning of drugs via L1000CDS^2^ has been employed in the context of different diseases: in Alzheimer’s disease (AD), to identify natural products that mimic or reverse AD-specific gene signatures in astrocytes based on cell line perturbations (repositioning e.g., the drug emetine based on liver cancer cell line HEPG2 data)^40^; in arthritis, where the antipsychotic drug amisulpride was repositioned based on similarities in gene expression patterns of perturbed cell lines (including the breast cancer cell line MCF7), with subsequent in vivo validation^41^; in asthma, where the drug lovostatin was identified based on perturbing the pulmonary adenocarcinoma cell line A549, and in COVID-19 where the drug candidate BML-259 was predicted based on perturbation of the lung adenocarcinoma cell line DV-90^42,43^.

Because the D + Q combination has been studied extensively in regard to cellular senescence and senolysis, and gene expression data of dasatinib- interventions are available online, the gene expression- based approach of repositioning was used to find candidate compounds that may replace dasatinib, to be used alone, or together with quercetin as a senolytic combination. Therefore, here we aimed to propose compounds that may show similar senolytic activity as dasatinib through computational drug repositioning, focusing on compounds found in dietary sources that could act as substitutes of dasatinib. More specifically, we aimed to (i) find studies about dasatinib that include publicly available gene expression data; (ii) identify differentially expressed genes (DEGs) associated with senescence and aging in these dasatinib intervention studies; (iii) search for dasatinib analogs, especially natural compounds, based on the DEGs related to dasatinib, employing the LINCS data and (iv) use the gene expression data underlying the repositioning to find hypotheses for potential senotherapeutic molecular mechanisms that dasatinib and its analogs may have in common. The molecular-mechanistic insights that we could derive from (ii) and (iv) suggest that the gene expression profile of dasatinib that we used for the repositioning is strongly linked to cellular senescence and apoptosis, as are the gene expression changes underlying the repositioning in case of the analogs (specifically, in case of piperlongumine). Thus, our approach should give us high confidence in their senotherapeutic effects, also in vivo in humans, by the analog itself or, potentially, by the analog in combination with quercetin.

## Results

In order to find repurposing candidates with similar gene expression profiles as dasatinib, we considered gene expression data from the GEO^44^ describing (1) the long-term effects of dasatinib (leading to dasatinib-resistance) in the AML cell line Kasumi-1, (2) transcriptomic differences in dasatinib-sensitive and dasatinib-resistant prostatic cancer cell lines, and (3) the effect of dasatinib-treatment on the breast cancer cell line MDA-MB-468 (see Table 1). For each of these, we describe the differentially regulated genes and their GO annotations. Then, we describe the results of the computational repurposing, and again we highlight the genes giving rise to the repurposing and their annotations.

### Genes regulated by dasatinib

#### Genes associated with aging and cellular senescence, and with biological processes associated with apoptosis, in the treated Kasumi-1 (AML) cell line

GEO accession GSE39073 entailed microarray gene expression profiles from AML- derived Kasumi-1 cells upon long-term treatment with dasatinib. The aim of these experiments was to study the effect of a 12-week exposure to dasatinib in leukemic cells, which usually triggers drug resistance and thus is a major problem for the treatment of patients with AML^45^. Here, the expression data from these experiments was re- analyzed to identify DEGs associated with regained drug-sensitivity, yielding 190 up- and 192 downregulated genes between both conditions (Supplementary Table 1). From these DEGs, eight genes (KYNU, FOS, ITGB2, PRKCD, BCL2, MPO, APP, TIMP2) were annotated with the GO term *aging* and one gene, PRKCD, with the term *cellular senescence* (Supplementary Table 2).

PRKCD (Protein kinase C) was upregulated with a log2 fold change (LFC) of 3.37 and it is a tumor suppressor protein and positive regulator of cell cycle progression; PRKCD may regulate apoptosis (see the NCBI Gene entry with ID 5580), and it plays a role in the regulation of senescence-induction in human diploid cells^46^. Also associated with cellular senescence, based on the literature, is the apoptosis regulator BCL2, which was upregulated with LFC = 2.62. BCL2 is an integral mitochondrial membrane protein that blocks apoptosis of e.g. lymphocytes (NCBI Gene ID 596). It is a pro-survival protein and a target of senolytics inducing apoptosis, and may influence human lifespan^11,47^.

Biological processes related to apoptosis were also enriched in the DEG list (adjusted p-value < 0.05) and included *cell death, programmed cell death, regulation of cell death, apoptotic process,* and *regulation of programmed cell death* (see Supplementary excel sheet “GO- gprofiler.5–23-22_AML”, for the enriched genes in the “intersections” column).

#### Genes associated with aging and cellular senescence, and with biological processes associated with apoptosis, in the dasatinib-sensitive prostatic cancer cell lines

GEO-Accession GSE9633 features baseline gene expression profiles of dasatinib-sensitive and dasatinib-resistant prostatic cancer cell lines. We identified 198 differentially expressed genes, with 138 upregulated and 51 downregulated genes between dasatinib-sensitive and dasatinib-resistant prostatic cancer cell lines. Six genes were annotated to the term *aging*: SERPINB5, CTSV, CLDN1, TGFBR2, CDKN2A and ASS1 (see also Supplementary Table 3 for the literature-derived association with *aging*).

SERPINB5, featured among the upregulated genes, is a tumor suppressor and senescence-associated marker^48,49^, the expression of which is linked to genotoxic and oxidative stress^39,42^. TGFBR2 was also upregulated in dasatinib-sensitive cell lines. This growth factor receptor may play a role in the interplay between cell survival and apoptosis in determining human lifespan^47^ as it is involved in the phosphorylation of transcription factors associated with proliferation, cell cycle arrest, immunosuppression and tumorigenesis (NCBI Gene ID 7048). Another upregulated gene is CDKN2A (p16) that encodes a well-established marker of cellular senescence^50^. Accumulation of p16-positive cells (suggested to be senescent) during adulthood negatively influences lifespan and promotes age-dependent changes and diseases in various organs and tissues^51^. Although the GO term “cellular senescence” was not enriched, the enrichment analysis showed that the cellular senescence pathway (KEGG accession ko04218) was enriched, and genes associated with this pathway were all upregulated, including some of the ones mentioned above (TGFBR2, TGFB2, HLA-A, CDKN2A, ZFP36L1, HLA-E, HLA-G, GADD45A, FOXO1, RRAS and GADD45B). Enriched biological processes also included processes associated with apoptosis (see Supplementary excel sheet “GO-gprofiler.5–23-22_PC- cancer” for enriched genes found in the “intersections” column), including *apoptotic process and positive regulation of apoptosis, programmed cell death* and *positive regulation of programmed cell death* (adjusted p-value < 0.05).

#### Genes associated with aging and cellular senescence, and with biological processes associated with apoptosis, in the MDA-MB-468 breast cancer cell line

The gene expression dataset with the accession PRJNA559155 includes expression profiles of the dasatinib-treated breast cancer cell line MDA-MB-468. Differential expression analysis resulted in 189 upregulated and 80 downregulated genes between dasatinib-treated- and control MDA-MB-468 cells. Among the differentially expressed genes, four of the genes were annotated with the term *aging*: CCL11, KRT25, RNF165 and SREBF1 (see Supplementary Table 4 for the literature-derived association with *aging*).

Among these genes, the CCL11 gene was most significantly downregulated with an LFC of − 9.78 in the dasatinib-treated cells and the effect may be mediated via HCK or other SRC kinases. CCL11 (also known as eotaxin-1) is considered to be an aging- and inflammation-associated plasma chemokine and a SASP factor^52,53^. It acts as an eosinophil chemoattractant, is associated with allergic responses and Th2 inflammatory disease, colon tumorigenesis^54^, and with cell migration in rheumatoid arthritis^55^. CCL11 is a putative biomarker for the prediction of severity and mortality of elderly patients with sepsis-induced myocardial injury^56^. SFREBF1 is a transcriptional activator needed for lipid homeostasis^57^ and possibly interacts with dasatinib via SRC-EP300. The GO term “cellular senescence” was not enriched, and none of the GO biological processes were associated with apoptosis (see Supplementary excel sheet “GO-gprofiler_5-23-22_BC”) (Table 2).

**Table 2:** List of datasets used in this study. AML: Acute myeloid leukemia.

Accession	Platform	Experiment	References
GSE39073	Affymetrix Human Gene 1.0 ST Array	Post-dasatinib exposure of AML cell line Kasumi-1 vs. untreated AML cell line Kasumi-1	^45^
GSE9633	Affymetrix Human Genome U133A 2.0 Array	Dasatinib-sensitive vs. dasatinib-resistant prostatic cancer cell lines	^58^
PRJNA559155	Illumina HiSeq 4000	Dasatinib-treated breast cancer cell line MDA- MB-468 vs. untreated MDA-MB-468 cells	^59^

#### Functional similarity of Biological Processes between the datasets

Using MegaGO, the similarity in gene ontology terms was assessed where the output is a value between 0 and 1 and describes relative similarity. Comparing biological processes from AML and PC- datasets results in a similarity score of 0.88, showing that the BPs are functionally related (highly similar would be a value > 0.9), whereas AML/PC and BC biological processes are not functionally similar (scores of 0.16 and 0.15, respectively).

### Repurposing of natural compounds by similarity to dasatinib

Candidate compounds were identified using the L1000CDS^2^ webtool, restricting the output to 50 predictions (corresponding to LINCS perturbations) that mimic or reverse the input signature (that is, the up- and downregulated genes identified from the differential expression analysis of dasatinib). *Reverse* matching was chosen for AML and prostate cancer (PC) datasets. In the AML dataset, post-dasatinib recovery is described, and we intent to counteract apoptotic inhibition and proliferative signaling in a senolytic fashion. In the case of the PC dataset, dasatinib resistance is described and senescence is intended to be counteracted, including the SASP, in a senomorphic fashion. Thus, to reverse these patterns,  downregulated genes of the query input are intersected with upregulated genes of the LINCS perturbation, and vice versa^39^. *Mimic* was chosen for the breast cancer (BC) dataset to mimic the effect of dasatinib, that is, the downregulation of inflammation and the SASP, which we intent to foster in a senomorphic fashion, and therefore,  downregulated genes of the query input are intersected with downregulated genes from the LINCS perturbation (and upregulated genes with upregulated genes). The selected compounds obtained from L1000CDS^2^ were labeled manually as *natural compounds* as appropriate (Table 3). In *reverse* mode, natural compounds from the AML-dataset were piperlongumine, parthenolide and curcumin on ranks 1, 20 and 40, respectively. Also, in *reverse* mode, natural compounds from the PC-dataset were piperlongumine and parthenolide on ranks 27 and 39. In *mimic* mode, natural compounds from the BC-dataset were phloretin and parthenolide on ranks 7 and 32.

**Table 3:** Selected natural compounds mimicking the treatment with dasatinib.

Rank	Dataset/ Score	Compound	Class of compound	Source
	Dataset GSE39073—Kasumi-1 cells, *reverse*			
1	0.0634	Piperlongumine	Amide alkaloid	*Piper longum*
20	0.0387	Parthenolide	Sesquiterpene lactone	*Tanacetum parthenium*
40	0.0352	Curcumin	Diarylheptanoid	*Curcuma longa*
	Dataset GSE9633—prostatic cancer cell lines, *reverse*			
27	0.071	Piperlongumine	Amide alkaloid	*Piper longum*
39	0.0656	Parthenolide	Sesquiterpene lactone	*Tanacetum parthenium*
	Dataset PRJNA559155—breast cancer cells, *mimic*			
7	0.0435	Phloretin (BRD-A36630025)	Dihydrochalcones	e.g. apples
32	0.0348	Parthenolide	Sesquiterpene lactone	*Tanacetum parthenium*

These compounds were identified using the L1000CDS^2^ tool. The rank is based on the overlap; the overlap is a score based on the intersection length between input DEGs and the signature DEGs divided by the effective input, i.e. the intersection-length between input genes and L1000 genes. The full tables are provided as supplemental tables.

Piperlongumine was the highest-ranking compound identified with the AML-dataset GSE39073, and the compound with the highest rank product^39^ considering all three datasets. The highest overlap (in terms of overlapping genes) was seen in piperlongumine- treated NOMO1 cells^39^; treatment dose was 10 μM. NOMO1 is an AML cell line^60^ just like the Kasumi-1 cell line. Overlapping genes of the *input upregulated* and the piperlongumine-based *signature downregulated* genes were ACSL1, ATP8B4, CTSG, EIF1AY, FLT3, HCK, KDM5D, LYZ, PLAC8, PRKCD, PTPN6, RNASE2, RPS6KA1, TNFRSF10B and TNS3. Overlapping genes of the *input downregulated* and the *signature upregulated* genes were DDAH1, FBXO21, SLC38A1 and TSPAN13 (Supplementary Table 5).

Enriched biological processes (adjusted p-value < 0.05) in the aggregate list of 19 genes include apoptosis-related processes (see Supplementary Table 6), e.g. *negative regulation of apoptotic process, intrinsic apoptotic signaling pathway, TRAIL-activated apoptotic signaling pathway, negative regulation of glial cell apoptotic process, negative regulation by symbiont of host apoptotic process* and *intrinsic apoptotic signaling pathway in response to oxidative stress.*

Piperlongumine was also found on rank 27 with the PC-dataset where PC3 cells were treated with 10 μM piperlongumine. PC3 is a dasatinib-resistant prostate cancer cell line^58^. *Input upregulated and signature downregulated* overlapping genes include AHNAK2, ALDH1A3, AREG, C3, CAPG, CST6, DDX60, FERMT1, ITGA3, KRT7, LAMA3, RAC2, RRAS, S100A2, TGFBR2 and ZBED2; one overlapping gene between *input downregulated and signature upregulated* genes was identified, which was LEF1 (see Supplementary Table 7).

Here, the biological process *positive regulation of apoptotic cell clearance* was associated with the C3 gene, and the RAC2 gene was associated with the process *engulfment of apoptotic cell clearance* (See Supplementary Table 8).

Curcumin was identified from the AML dataset, on rank 40, where PL21 cells were treated with 48 μM curcumin^39^. PL21 is also an AML cell line^61^. Overlapping genes of the *input upregulated* and the curcumin *signature downregulated* genes were ADCY7, AHNAK, ATP8B4, BEX1, CXCR4, DDX3Y, EIF1AY, EPB41L3, KDM5D, PRKCD, RASSF2 and VIM. One gene overlapped with the *input downregulated* genes and the *signature upregulated* genes, which was MEST. Enriched apoptosis-associated biological processes (p-value < 0.05) included *regulation of glial cell apoptotic process* and *intrinsic apoptotic signaling pathway in response to oxidative stress*.

Phloretin is a dihydrochalcone flavonoid found in fruits such as apples, kumquat, pear, strawberry and in vegetables^62^. Phloretin appeared on rank 7 from the expression signature of BC-dataset PRJNA559155. One gene overlapped with the *input upregulated* and *signature upregulated* genes, which was DYRK3. Overlapping genes with *input downregulated* and *signature downregulated* genes were ASPM, CALD1, IFITM2 and LDLR.

Parthenolide is a sesquiterpene lactone of the chemical class of terpenoids^63^ and was identified with L1000 using all three datasets, though at low ranks in all of these: on rank 20 with the AML- dataset, on rank 39 with the PC-dataset, and on rank 32 with the BC-dataset (Table 3).

## Discussion

In this study, we identified natural senotherapeutic compounds, and one compound, piperlongumine, with senolytic activity by using a bioinformatics workflow based on publicly available gene expression profiles after treatment of cells with dasatinib and natural compounds. Using L1000CDS^2^ we obtained lists of compounds that induce similar gene expression profiles as compared to the input gene lists describing the action of dasatinib. Natural compounds were curated manually, and we identified four natural candidate-compounds, all of which are found in common foods and all of which have already been under investigation for their anti-inflammatory, anti-cancer or anti-aging effects: piperlongumine, phloretin, curcumin and parthenolide.

The most promising candidate, piperlongumine is a known natural senolytic compound that was found based on the genes differentially expressed between dasatinib-treated Kasumi-1 (AML-dataset GSE39073) and untreated cells, which show an overlap with the L1000 dataset of the piperlongumine-treated AML cell line NOMO1. This overlap of DEGs featured an enrichment in genes and processes involved in apoptosis, including *intrinsic apoptotic signaling pathway in response to oxidative stress* and *intrinsic apoptotic signaling pathway*.

Since most senotherapeutics act cell type specific in the context of senescence^64^, the cell types used to generate the gene expression datasets used in our analyses need to be taken into consideration when interpreting the obtained results. The fact that Kasumi-1 cells as well as NOMO1 cells both are derived from AML suggests that piperlongumine may induce similar gene expression effects in AML cancer cells as dasatinib but it may not have the same effect on senescent non-cancer cells. However, piperlongumine is one of the few natural compounds shown to selectively kill senescent cells, that is, human WI-38 fibroblasts made senescent by ionizing radiation, replicative exhaustion or by expression of the Ras oncogene^8,29^ and therefore, it is a promising repurposing candidate in our context.

Piperlongumine is a natural compound with a strong safety record: In vivo, the safety and therapeutic efficacy of piperlongumine was tested in a mouse xenograft model of thyroid cancer^65^. After intraperitoneal injection of 10 mg/kg piperlongumine, no changes in body weight or pathological alterations in liver, kidney and tumor tissues were reported, and no significant infiltration of immune cells in those tissues was found, while the piperlongumine-treated mice had significantly lower tumor volumes compared to control mice^65^. Furthermore, piperlongumine has selective toxicity toward cancer cells and senescent cells, but does not induce significant toxicity in non-senescent, non-cancerous cells^29,66^, including peripheral blood T cells^67^. Incubating senescent WI human fibroblasts for 72 h leaves 30% of the senescent cells viable^29^. In this study, the same 10 μM dose-regimen was used as has been used for the L1000 data. When combining piperlongumine with ABT-263 (navitoclax, a potent BCL2 inhibitor)^29^, a synergistic effect was observed, killing almost all senescent cells; the authors suggested that piperlongumine eradicated the subpopulation of senescent cells that was resistant to ABT-263^29^. While BCL2 family proteins are thought to be partially responsible for the ability of senescent cells to resist apoptosis, and BCL2/BCL2L1/BCL2L2 inhibitors are effective senolytic drugs (e.g. ABT-263)^68^, there is a concern that BCL2 inhibitors may also trigger toxic effects, such as thrombocytopenia and neutropenia^69^.

Data on the mode of action of piperlongumine in general, and specifically on how it induces apoptosis in cancer cells is available from a number of studies (e.g.^65,70^.). Senescent cells and cancer cells share some pro-survival pathways, e.g., active DNA damage responses^71^, high metabolic activity including increased glycolysis^72^, and the reliance on dependence receptors to resist apoptosis^73^. Data from these studies, including our results from L1000 (especially the overlapping genes associated with apoptosis) based on repurposing dasatinib-associated expression data, thus suggest piperlongumine-induced apoptosis in senescent cells^8,29^.

In more detail, piperlongumine has been shown to kill senescent fibroblasts without the induction of reactive oxygen species^29^, though it was later demonstrated that it inhibits the OXR1 (oxidation-resistance 1) protein that in turn leads to the expression of antioxidant genes. OXR1 is upregulated in senescent human WI38 fibroblasts and thus it is a proposed senolytic target^9^. When piperlongumine binds to OXR1 (see Supplementary Fig. 1), the protein is degraded, leading to increased production of reactive oxygen species in senescent cells, mediated by low or zero levels of antioxidant genes such as heme oxygenase 1 (HMOX1), glutathione peroxidase 2 (GPX2) and catalase, presumably due to missing/reduced OXR1. Then, senescent cells are more susceptible to oxidative stress, leading to their apoptosis^9,74^. Of note, GPX2 (or glutathione) is a major hydrogen peroxide and organic hydroperoxide scavenger (also regulated by NRF2), induced by e.g. cigarette smoke^75^.

Piperlongumine has also been shown to interfere with T-cell differentiation and is considered to be a selective immunosuppressant^67^, partly, again by a pro-oxidative action, here due to intracellular depletion of glutathione levels^74^. This was linked to the inhibition of the transcription factors RORC (RORγt), HIF1A and STAT3, resulting in reduced production of IL22, IL17A, IL17F, and subsequent inhibition of Th17- differentiation, but not of regulatory Th1 and Th2 cells (Tregs), along with reduced expression of CD69 and CD35 expression markers^67,74,76^. This is interesting and important, because the Th17/Treg ratio increases during aging, and increasing Th17/Treg imbalance possibly contributes to an altered pro-inflammatory/ anti-inflammatory immune response and thus indicates a higher risk to develop inflammatory diseases with increasing age^77^.

In our analyses, we specifically focused on overlapping genes between the dasatinib-associated gene expression changes and piperlongumine-treated cells from the L1000 database, looking for genes related to aging, senescence and apoptosis. One of the downregulated genes in the piperlongumine-based signature, overlapping with the AML-cell line data (Supplementary Table 5), is PTPN6 (also known as SHP-1), a tyrosine phosphatase that has been shown to interfere with cellular senescence via p16 signaling, and was proposed to regulate senescence in nasopharyngeal carcinoma cells^78^. Two other downregulated overlapping genes were FLT3 and HCK, both enriched in the biological process *apoptotic process*, and additionally in the pathway *FLT3 signaling through SRC family kinases* (HAS-9706374). FLT3 and HCK are described as attractive targets for cancer therapy. Experimentally, FLT3 inhibition led to apoptosis in FLT3 positive AML cells^79^, and dasatinib was shown to reverse induced resistance to FLT3-inhibition in the treatment of AML^80^. HCK has an important role in the production of TNF and IL-6, enhances the secretion of growth factors, and targeting HCK has been proposed to alleviate excessive inflammation^81,82^. In the overlapping genes between the PC-data and piperlongumines’ effects as known from L1000, the CS3 gene was positively associated with the regulation of apoptotic cell clearance (see supplementary excel sheet overlap_PL_PC3_PC-dataset), and it is downregulated in response to piperlongumine in PC cells. Overall, our data suggests that the mechanisms of piperlongumine are related to senescence and apoptosis, considering the overlapping genes and the enriched biological processes just described. Moreover, the available evidence for the upstream mechanisms leading to apoptosis by piperlongumine based on published experiments also feature apoptosis as its downstream effect, consistent with the high-throughput transcriptomics data we investigated.

Other identified compounds were phloretin, parthenolide and curcumin. We did not conduct a detailed investigation of published mechanisms as in case of our top hit piperlongumine, but we note that we again found associations with apoptosis based on our data, in particular in case of curcumin. Additional information on pharmacokinetics and bioavailability of the identified compounds is provided in the Supplementary Material.

Lastly, it is important to acknowledge the limitations of our study. We used a computational approach, assuming that similarities in gene expression profiles between dasatinib and natural compounds in vitro imply comparable senolytic/senomorphic activities, despite the complexity of biological systems and the potential for divergent mechanisms of action in vivo. Further, the accuracy of our findings is dependent on the datasets used. We relied on existing gene expression data, introducing potential biases related to experimental conditions and cell line specificity. From the datasets, including the PC-dataset may be controversial since dasatinib has not been used to treat the cell lines directly, but rather the difference in expression profiles between dasatinib-sensitive and dasatinib-resistance cell lines was assessed. In addition, it is essential to further confirm and evaluate the senolytic and senomorphic potential of the identified compounds in cell lines, animal models, or organoids, alongside safety assessments prior to human studies. Exploring alternative analytical methods, such as knowledge graphs or large language models could also yield deeper insights and potentially reveal mechanisms or interactions not evident through traditional analyses.

In summary, we used a computational approach to identify four natural compounds that may mimic dasatinib based on gene expression data. While our findings are promising, cautious interpretation is necessary and subsequent experimental validation and methodological diversification is crucial to fully ascertain the therapeutic potential and safety of these compounds.

## Methods

### Methods overview

**Figure 1:**
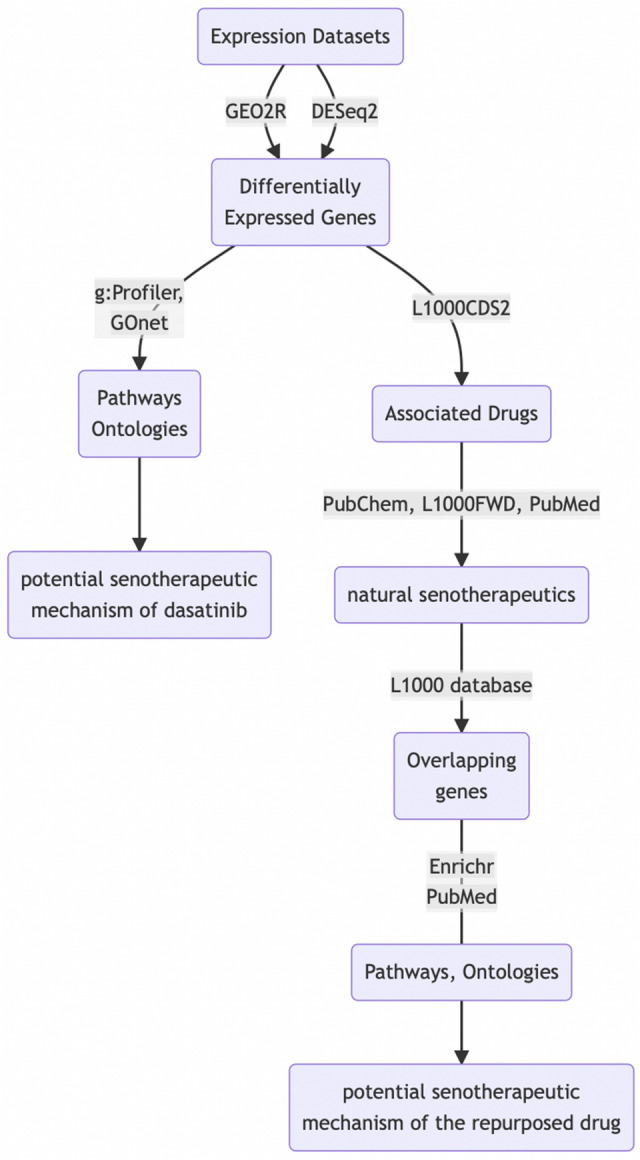
Graphical abstract showing the workflow to find natural candidate compounds with similar senolytic activity as dasatinib from expression data.

The following diagram depicts the workflow, based on bioinformatics methods and public data, to identify candidate compounds with similar senolytic activity as dasatinib (Fig. 1).

### Expression data

Searches for gene expression studies about drug interventions with dasatinib were conducted in the European Nucleotide Archive (ENA), the European Genome-phenome archive, the Gene Expression Omnibus (GEO) and Google Datasets (https://datasetsearch.research.google.com). Only RNA-seq and microarray datasets were considered. The search keywords included: “aging”, “senescence”, “inflammation”, “cancer”, “apoptosis”, “SASP” and “senolysis”, and they were used in combination with “dasatinib”. We found nine datasets from which the following three are subject of this paper (Table 2): dataset GSE39073, a microarray dataset containing gene expression profiles of the acute myeloid leukemia (AML) cell line Kasumi-1 subjected to long-term treatment of dasatinib^45^; GSE9633, microarray data from experiments related to dasatinib-sensitive and dasatinib-resistant prostatic cancer cell lines (D-sensitive cell lines: 22Rv, WPMY1, VCaP, MDAPCa2b, PWR1E; D-resistant cell lines: PC3, DU145, LNCaP, HPV7, HPV10, RWPE1, RWPE2, NB11, W99, DUCaP; the strength of this dataset lies in its use of more than one cell line)^58^; and PRJNA559155, with RNA-seq expression data from breast cancer cell line MDA-MB-468 exposed to either dasatinib, salinomycin, or a combination of both^59^.

The remaining datasets were excluded for reasons described in the following. Drug repositioning with gene expression signatures did not result in the identification of natural substances at the chosen cutoffs (dataset GSE59357); gene expression analysis did not result in significantly differentially expressed genes (dataset GSE69395); single-cell RNA-seq experiments cannot be directly compared against pooled cell data as stored in LINCS (accession GSE161340); cells were neither exposed to dasatinib, nor sensitivity/resistance to dasatinib was assessed as part of the experiment (datasets EGAD00001001016 and GSE14746); too few RNA-seq reads were obtained after quantification of single-end reads (-r) from fastq-files in mapping-based mode (i.e. salmon quant) to the human transcriptome using salmon^83^ (dataset PRJNA613485).

### Expression analysis

Differential expression analysis of the two microarray datasets was done using the web program GEO2R (accessed May 6th 2021). This program relies on GEOquery (version 2.58.0) for data retrieval, and on the R package limma^84^ (version 3.46.0) for the assessment of differential expression. Accordingly, these methods were applied for the selected microarray datasets (accessions GSE39073 and GSE9633)*.* In turn, raw RNA sequencing reads from the accession PRJNA559155 were downloaded from the ENA and mapped to the human transcriptome (GENCODE release 38) using salmon v1.4.0^83^. Differential expression analysis was then performed using DESeq2 version 1.32.0^85^, for the comparison between the treatment (MDA-MB-468 cells exposed to dasatinib), and control (untreated MDA-MB-468 cells) groups. The obtained sets of differentially expressed genes were then filtered according to expression fold changes and adjusted p-values as in Supplementary Table 1.

### Functional analyses

Initial Gene ontology and KEGG pathway^86^ enrichments were obtained with the g:profiler webtool (^87^; accessed 2021–11-25 and 2022–05-23) and GOnet (https://tools.dice-database.org/GOnet/) was used to perform gene annotation analysis to find genes annotated with *aging* and *senescence*^88^. Enrichr (https://maayanlab.cloud/Enrichr/) was used to identify biological processes of the overlapping genes^89^ (version 2021, accessed 2022–05-14). MegaGO (accessed 2023–01-13) was employed to obtain a score describing the similarity in biological processes between the experiments. Finally, the NCBI gene database (https://www.ncbi.nlm.nih.gov/gene/), the human gene database GeneCards (https://www.genecards.org) and PubMed were used to provide gene-associated annotation information.

### Drug repurposing

L1000CDS^2^, a webtool that processes expression-perturbation data from the L1000 resource with a method that prioritizes small-molecule signatures that either mimic or reverse an input gene expression signature, was used for compound identification^39^. When submitting the “input” (up- and downregulated genes obtained from the differential expression analysis) to L1000CDS^2^, 50 predictions (of small molecules/chemicals, characterized as perturbators of gene expression in cell lines) ranked by their overlap with the “input” signature were considered as output. Each prediction (corresponding to a perturbation) comes with seven items of information, provided in a table. This includes the rank (which is based on the overlap), the overlap (a value based on the intersection length between the input DEGs and the signature DEGs divided by the effective input, i.e. the intersection-length between input genes and L1000 genes); a venn diagram (a schematic representation of the (mimicked or reversed) overlap of the input signature and L1000 signature), the perturbation and its associated the cell line, dose and time, the list of overlapping genes, the predicted target genes of the perturbation, and the signature of the target/hit^39^. Upregulated and downregulated genes were used as input separately, ordered by descending log2 fold changes. For the accession PRJNA559155, *mimic* mode (i.e. *mimicking* the effect of the drug by reproducing the gene expression changes associated with dasatinib), was chosen, and for datasets GSE9633 and GSE39073 *reverse* mode (i.e. *reversing* the disease phenotype that is susceptible to dasatinib) was chosen. The resulting lists of compounds were then manually curated, looking up each compound in the PubChem database, and in PubMed, to identify natural plant metabolites.

The tabulated L1000CDS^2^ results are available online via *permanent* URLs:

- AML-cell line (GSE39073, reverse): https://maayanlab.cloud/L1000CDS2/#/result/628b901ab94e3c005691571e
- PC-cell line (GSE9633, reverse): https://maayanlab.cloud/L1000CDS2/#/result/619bb34fd99ec600506d5e20
- BC-cell line (PRJNA559155, mimic): https://maayanlab.cloud/L1000CDS2/#/result/619f82f7d99ec600506d6086

### Supplementary Information

Supplementary Information 1.

Supplementary Information 2.

## Data Availability

The datasets analyzed in this study are available in the GEO and ENA repositories via the following links: GSE9633: https://www.ncbi.nlm.nih.gov/geo/query/acc.cgi?acc=GSE9633 ; GSE39073: https://www.ncbi.nlm.nih.gov/geo/query/acc.cgi?acc=GSE39073 ; PRJNA559155: https://www.ebi.ac.uk/ena/browser/view/PRJNA559155.
