# Supplementary material for: Computational identification of natural senotherapeutic compounds that mimic dasatinib based on gene expression data
Source: Sci Rep. 2024 Mar 15;14:6286. doi: 10.1038/s41598-024-55870-4 (PMC10943199; doi:10.1038/s41598-024-55870-4)
Supplement: Supplementary file 1 — Supplementary Information 1. [file 41598_2024_55870_MOESM1_ESM.pdf]

Computational identification of natural senotherapeutic compounds that mimic dasatinib based on gene expression data

Franziska Meiners[1], Burkhard Hinz[2], Lars Boeckmann[3], Riccardo Secci[1], Salem Sueto[1], Lars Kuepfer[4], Georg Fuellen[1*], Israel Barrantes[1]

[1] Institute for Biostatistics and Informatics in Medicine and Ageing Research Rostock University Medical Center, Rostock, Germany

[2] Institute of Pharmacology and Toxicology, Rostock University Medical Center, Rostock, Germany

[3] Clinic and Policlinic for Dermatology and Venerology, University Medical Center Rostock, Strempelstr. 13, 18057, Rostock, Germany

[4] Institute for Systems Medicine with Focus on Organ Interaction, University Hospital RWTH Aachen, Aachen, Germany.

Reference is made herein to the supplementary excel-sheet **dasatinib_compid_V03 Supplem_Material.xlsx**

Correspondence

Georg Fuellen, Institute for Biostatistics and Informatics in Medicine and Ageing Research, University Medical Center Rostock, Rostock
Email: fuellen@uni-rostock.de

Journal: Scientific Reports

Number of Supplementary Tables: 8

Number of Supplementary Figures: 1

Number of Supplementary Texts: 1

Supplementary Table Legends

Supplementary Table 1: Number of upregulated/ downregulated genes of each of the datasets

Supplementary Table 2: Genes annotated with the biological process *aging.* PRKCD was annotated also with the biological process *cellular senescence*; DEGs from AML-dataset GSE39073 were used. Genes were identified with the GOnet-webtool (https://tools.dice-database.org/GOnet/) with analysis type "GO-term annotation". LFC: log2 fold change.

Supplementary Table 3: Genes annotated with the biological process *aging*, DEGs from prostate-cancer (PC) dataset GSE9633. Genes were identified with the GOnet-webtool (https://tools.dice-database.org/GOnet/) with analysis type "GO-term annotation". LFC: log2 fold change.

Supplementary Table 4: Genes annotated with the biological process *aging*, DEGs from breast-cancer dataset PRJNA559155 were used. Genes were identified with the GOnet-webtool (https://tools.dice-database.org/GOnet/) with analysis type "GO-term annotation". LFC: log2 fold change.

Supplementary Table 5: Overlapping genes between the up-(and down)regulated genes of AML-dataset GSE39073, and down-(and up)regulated genes of piperlongumine-induced expression changes from the L1000 database.

Supplementary Table 6: Enriched biological processes associated with *apoptosis* of overlapping genes between the up- (and down)regulated

genes of AML-dataset GSE39073, and down (and up)regulated genes of piperlongumine-induced expression changes from the L1000 database. Obtained from enrichr version 2021. The complete table of enriched biological processes is available in the excel-sheet: overlapGO_NOMO1_PL_AML-05-15-21

Supplementary Table 7: Overlapping genes between the up- (and down)regulated genes of PC-dataset GSE9633, and down (and up) genes of piperlongumine-induced expression changes from the L1000 database.

Supplementary Table 8: Enriched biological processes associated with *apoptosis* of overlapping genes between the up- (and down)regulated genes of PC-dataset GSE9633, and down (and up) genes of piperlongumine-induced expression changes from the L1000 database. Obtained from enrichr version 2021. The complete table of enriched biological processes is available in the excel-sheet; overlapGO_PL_PC3_PC-dataset

Supplementary Tables

Supplementary Table 1: Number of upregulated/ downregulated genes of each of the datasets

Dataset	Adjusted p-value	Upregulated DEGs	Downregulated DEGs
GSE39073, LFC = 2	< 0.01	190	192
GSE9633 LFC = 2	< 0.05	138	51
PJRNA559155 LFC = 1.5	< 0.05	109	80

Supplementary Table 2: Genes annotated with the biological process *aging.* PRKCD was annotated also with the biological process *cellular senescence*; DEGs from AML-dataset GSE39073 were used. Genes were identified with the GOnet-webtool (https://tools.dice-database.org/GOnet/) with analysis type "GO-term annotation". LFC: log2 fold change.

Gene	LFC	association with aging	Reference
KYNU	5.16	Knockout in C. elegans resulted in lifespan extension of >20%	[1]

FOS	3.58	associated with ovarian aging, role in activated human T-cells	[1,2]
PRKCD	3.37	tumor suppressor protein, cell cycle regulator, apoptosis regulation. Association with senescence-induction in human diploid cells	[3]
ITGB2	2.73	probable role in ovarian aging, higher expression in ovaries from old mice	[4]
BCL2	2.62	senolytic BCL2 inhibition leads to apoptosis of senescent cells, may influence human lifespan	[5,6]
MPO	-3.03	aging, immune cells. High MPO levels can be reduced by a calorie-restricted diet	[7]
APP	-2.79	protective response to aging-induced inflammation in endothelial cells	[8]
TIMP2	-2.36	associated with synaptic plasticity and cognition in aged mice	[9]

Supplementary Table 3: Genes annotated with the biological process *aging*, DEGs from prostate-cancer (PC) dataset GSE9633. Genes were identified with the GOnet-webtool (https://tools.dice-database.org/GOnet/) with analysis type "GO-term annotation". LFC: log2 fold change.

Gene	LFC	association with aging	Reference
SERPINB5	6.66	tumor suppressor and senescence-associated biomarker	[10,11]
CTSV	4.06	Matrix-degrading protease associated with skin aging	[12]
CLDN1	4.01	Polymorphisms are associated with age (55 or older) in breast cancer patients	[13,14]
TGFBR2	3.61	Regulation of cell survival and apoptosis with a potential role in human longevity	[5]
CDKN2A	3.12	senescence-associated marker	[15]
ASS1	3.06	Association with Alzheimer's disease	[16]

Supplementary Table 4: Genes annotated with the biological process *aging*, DEGs from breast-cancer dataset PRJNA559155 were used. Genes were identified with the GOnet-webtool (https://tools.dice-database.org/GOnet/) with analysis type "GO-term annotation". LFC: log2 fold change.

Gene	LFC	association with aging	Reference
CCL11	-9.78	SASP factor aging- and inflammation associated plasma cytokine	[17]
KRT25	-7.64	hair greying	[18]
RNF165	3.38	enhances BMP-smad signalling and mediates motor axon extension, knockout associated with premature death in mice	[19]
SREBF1	1.83	regulates lipid homeostasis, possibly an aging-associated transcription factor	[20,21]

Supplementary Table 5: Overlapping genes between the up-(and down)regulated genes of AML-dataset GSE39073, and down-(and up)regulated genes of piperlongumine-induced expression changes from the L1000 database.

Input up/ signature down	input down/ signature up
ACSL1	DDAH1
ATP8B4	FBXO21
CTSG	SLC38A1
EIF1AY	TSPAN13
FLT3	
HCK	
KDM5D	
LYZ	
PLAC8	
PRKCD	
PTPN6	
RNASE2	
RPS6KA1	
TNFRSF10B	
TNS3	

Supplementary Table 6: Enriched biological processes associated with *apoptosis* of overlapping genes between the up- (and down)regulated genes of AML-dataset GSE39073, and down (and up)regulated genes of piperlongumine-induced expression changes from the L1000 database. Obtained from enrichr version 2021. The complete table of enriched biological processes is available in the excel-sheet: overlapGO_NOMO1_PL_AML-05-15-21

Term	Adjusted P-value	Genes
regulation of apoptotic process (GO:0042981)	1.35E-02	HCK;FLT3;RPS6KA1; TNFRSF10B;PTPN6
negative regulation of apoptotic process (GO:0043066)	1.93E-02	HCK;PRKCD;RPS6KA 1;CTSG
intrinsic apoptotic signaling pathway (GO:0097193)	4.43E-02	PRKCD;TNFRSF10B
regulation of glial cell apoptotic process (GO:0034350)	4.43E-02	PRKCD
TRAIL-activated apoptotic signaling pathway (GO:0036462)	4.43E-02	TNFRSF10B
modulation by symbiont of host apoptotic process (GO:0052150)	4.43E-02	CTSG
negative regulation of glial cell apoptotic process (GO:0034351)	4.43E-02	PRKCD
negative regulation by symbiont of host apoptotic process (GO:0033668)	4.43E-02	CTSG

intrinsic apoptotic signaling pathway in response to oxidative stress (GO:0008631)	4.64E-02	PRKCD

Supplementary Table 7: Overlapping genes between the up- (and down)regulated genes of PC-dataset GSE9633, and down (and up) genes of piperlongumine-induced expression changes from the L1000 database.

Input up/ signature down	input down/ signature up
AHNAK2	LEF1
ALDH1A3	
AREG	
C3	
CAPG	
CST6	
DDX60	
FERMT1	
ITGA3	
KRT7	
LAMA3	
RAC2	
RRAS	
S100A2	
TGFBR2	
ZBED2	

Supplementary Table 8: Enriched biological processes associated with *apoptosis* of overlapping genes between the up- (and down)regulated genes of PC-dataset GSE9633, and down (and up) genes of piperlongumine-induced expression changes from the L1000 database. Obtained from enrichr version 2021. The complete table of enriched biological processes is available in the excel-sheet; overlapGO_PL_PC3_PC-dataset

Term	Adjusted P-value	Genes
engulfment of apoptotic cell (GO:0043652)	4.26E-02	RAC2
positive regulation of apoptotic cell clearance (GO:2000427)	4.17E-02	C3
regulation of apoptotic cell clearance (GO:2000425)	4.17E-02	C3

Supplementary Figures

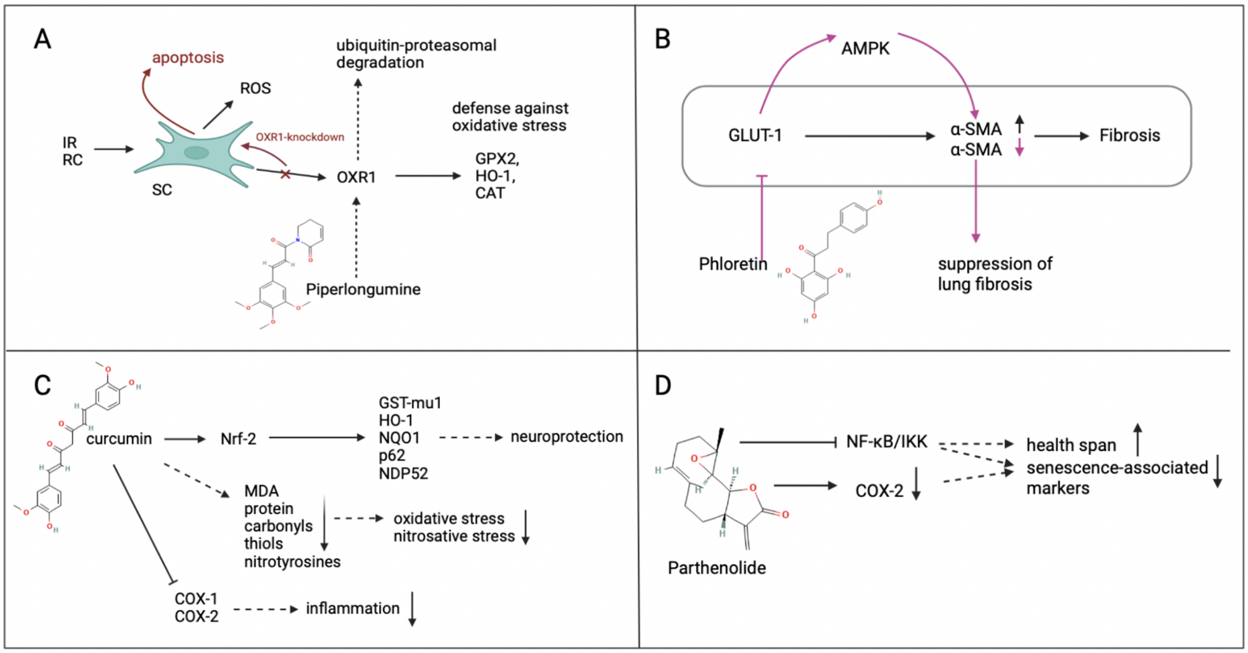

Supplementary Figure. 1. Proposed models of how the identified compounds may interact with senescence/aging. (A), potential mechanisms of senolytic action of Piperlongumine. As concluded by X. Zhang et al. (2018) piperlongumine directly binds to OXR1, suppressing the expression of antioxidant genes which leads to apoptosis of senescent fibroblasts [22]. (B) Phloretin is a potent SLC2A1 (GLUT-1) inhibitor. According to experiments conducted by Cho et al., (2017), its administration leads to activation of AMP-Kinase and reduces expression of α-smooth muscle actin in lung fibroblasts leading to suppression of lung fibrosis in aged mice [23]. (C) Proposed mechanism of anti-inflammatory, neuroprotective and anti-aging effects of curcumin as identified by Park et al., 2021 [24]. (D) Proposed targets of Parthenolide and potential age-related effects according to Zhang et al. (2021): Parthenolide inhibits NF-κB and IBK-Kinase (IKK)-complex, and downregulates COX-2 leading to reduction of SASP-factors and an increase in health span in mice model [25]. SC: senescent cell; RO: reactive oxygen species; IR: ionizing radiation; RC: replicative senescence.

Supplementary Texts

Pharmacokinetics and bioavailability of the identified natural compounds

Piperlongumine

Piperlongumine (piperlartine) is a biologically active amide alkaloid. Piperlongumine is metabolized via cytochrome P450-mediated oxidation at the lactam ring and the trimethlyphenyl residue [26]. Moreover, piperlongumine competitively inhibits CYP1A2 in human liver microsomes [27] and suppresses the metabolism of docetaxel via cytochrome P450 and P-glycoprotein [28]. Interestingly, very low maximum plasma concentrations with a high volume of distribution at steady state were recorded after intravenous injection of piperlongumine into mice [29]. In contrast, a significant improvement in bioavailability was registered in the same study after oral administration of [29].

Preclinical studies show that concomitant treatment of rats with piperlongumine leads to a 1.68-fold increase in the oral bioavailability of docetaxel, suggesting sufficient bioavailability of the alkaloid [28]. However, there are no preclinical or clinical studies in which the oral bioavailability of piperlongumine has been calculated (for review see [30]). The formulation in nanoemulsions leads to improved dissolution and cellular permeability of piperlongumine. Accordingly, nanoemulsions loaded with piperlongumine showed a 1.5-fold increase in oral bioavailability compared to free piperlongumine [29].

Parthenolide

Parthenolide is a germacran-type sesquiterpene lactone whose low bioavailability is a major limitation in clinical use (for review see [31]). An improved water solubility and oral bioavailability of about 70% exhibits dimethylaminopathenolide, a hydrophilic parthenolide analog that has been tested in clinical phase I for the treatment of acute myeloid leukemia [32].

Curcumin

Curcumin is a natural polyphenol that also has pharmacokinetic deficits due to its low oral bioavailability [33]. In fact, curcumin is poorly absorbed after oral ingestion and can therefore only be detected in the blood of test subjects in individual cases, while the corresponding glucuronide and sulphate conjugates can be determined in all of them [34]. In addition, curcumin is extensively converted to various phase I and phase II metabolites in the liver and the intestine [33]. Approaches to improve the pharmacokinetics have been developed and tested accordingly. These range from micronized curcumin [35] to formulations with piperine, which inhibits the intestinal and hepatic metabolism of curcumin [36].

Phloretin

Due to its low water solubility, phloretin, a dihydrogen chalcone flavonoid, has a poor bioavailability of about 8.67% after oral administration in rats [37]. In addition, phloretin undergoes rapid elimination. For this reason, phlorizin, a phloretin glycoside, is often used as a substitute for phloretin in commercial products. The low absorption and bioavailability of phloretin can be increased by using self-nanoemulsions, liposome and microemulsion formulations (for review see [38]. The bioavailability of phloretin can also be increased by the coadministration of P-glycoprotein and MRP2 inhibitors [37] or modified dosage forms [39].

In summary, the low oral bioavailability proves to be a challenge of the substances presented. However, as shown, bioavailability can be increased in many ways (e.g. nanoemulsions, micronized forms, hydrophilic analogues, coadministration of metabolism inhibitors), so that sufficient therapeutic value of these natural substances can still be accomplished if bioavailability is considered a problem.

References

1. Jiang Z-X, Wang Y-N, Li Z-Y, Dai Z-H, He Y, Chu K, Gu J-Y, Ji Y-X, Sun N-X, Yang F, Li W. Correction: The m6A mRNA demethylase FTO in granulosa cells retards FOS-dependent ovarian aging. Cell Death & Disease. 2021; 12: 1114.

2. Whisler RL, Chen M, Beiqing L, Carle KW. Impaired induction of c-fos/c-jun genes and of transcriptional regulatory proteins binding distinct c-fos/c-jun promoter elements in activated human T cells during aging. Cellular Immunology. 1997; 175: 41–50.

3. Katakura Y, Udono M, Katsuki K, Nishide H, Tabira Y, Ikei T, Yamashita M, Fujiki T, Shirahata S. Protein kinase C delta plays a key role in cellular senescence programs of human normal diploid cells. Journal of Biochemistry. 2009; 146: 87–93.

4. Ma L, Lu H, Chen R, Wu M, Jin Y, Zhang J, Wang S. Identification of Key Genes and Potential New Biomarkers for Ovarian Aging: A Study Based on RNA-Sequencing Data. Frontiers in Genetics. 2020; 11: 590660.

5. Ukraintseva S, Duan M, Arbeev K, Wu D, Bagley O, Yashkin AP, Gorbunova G, Akushevich I, Kulminski A, Yashin A. Interactions Between Genes From Aging Pathways May Influence Human Lifespan and Improve Animal to Human Translation. Frontiers in Cell and Developmental Biology. 2021; 9: 692020.

6. Zhu M, Meng P, Ling X, Zhou L. Advancements in therapeutic drugs targeting of senescence. Therapeutic Advances in Chronic Disease. 2020; 11: 204062232096412.

7. Son TG, Zou Y, Yu BP, Lee J, Chung HY. Aging effect on myeloperoxidase in rat kidney and its modulation by calorie restriction. Free Radical Research. 2005; 39: 283–9.

8. d'Uscio LV, Katusic ZS. Endothelium-specific deletion of amyloid-β precursor protein exacerbates endothelial dysfunction induced by aging. Aging. 2021; 13: 19165–85.

9. Castellano JM, Mosher KI, Abbey RJ, McBride AA, James ML, Berdnik D, Shen JC,

Zou B, Xie XS, Tingle M, Hinkson IV, Angst MS, Wyss-Coray T. Human umbilical cord plasma proteins revitalize hippocampal function in aged mice. Nature. 2017; 544: 488–92.

10. Bascones-Martínez A, López-Durán M, Cano-Sánchez J, Sánchez-Verde L, Díez-Rodríguez A, Aguirre-Echebarría P, Álvarez-Fernández E, González-Moles MA, Bascones-Ilundain J, Muzio LL, Campo-Trapero J. Differences in the expression of five senescence markers in oral cancer, oral leukoplakia and control samples in humans. Oncology Letters. 2012; 3: 1319–25.

11. Sheng S, Carey J, Seftor EA, Dias L, Hendrix MJ, Sager R. Maspin acts at the cell membrane to inhibit invasion and motility of mammary and prostatic cancer cells. Proceedings of the National Academy of Sciences. 1996; 93: 11669–74.

12. Panwar P, Hedtke T, Heinz A, Andrault P-M, Hoehenwarter W, Granville DJ, Schmelzer CEH, Brömme D. Expression of elastolytic cathepsins in human skin and their involvement in age-dependent elastin degradation. Biochimica Et Biophysica Acta General Subjects. 2020; 1864: 129544.

13. Blanchard AA, Ma X, Dueck KJ, Penner C, Cooper SC, Mulhall D, Murphy LC, Leygue E, Myal Y. Claudin 1 expression in basal-like breast cancer is related to patient age. BMC cancer. 2013; 13: 268.

14. Katoh M. Multi-layered prevention and treatment of chronic inflammation, organ fibrosis and cancer associated with canonical WNT/β-catenin signaling activation (Review). International Journal of Molecular Medicine. 2018; 42: 713–25.

15. Childs BG, Baker DJ, Kirkland JL, Campisi J, Deursen JM. Senescence and apoptosis: dueling or complementary cell fates? EMBO reports. 2014; 15: 1139–53.

16. Prasad KN. Simultaneous activation of Nrf2 and elevation of antioxidant compounds for reducing oxidative stress and chronic inflammation in human Alzheimer's disease. Mechanisms of Ageing and Development. 2016; 153: 41–7.

17. Cameron AR, Morrison VL, Levin D, Mohan M, Forteath C, Beall C, McNeilly AD, Balfour DJK, Savinko T, Wong AKF, Viollet B, Sakamoto K, Fagerholm SC, et al. Anti-Inflammatory Effects of Metformin Irrespective of Diabetes Status. Circulation Research. 2016; 119: 652–65.

18. Choi HI, Choi GI, Kim EK, Choi YJ, Sohn KC, Lee Y, Kim CD, Yoon TJ, Sohn HJ, Han SH, Kim S, Lee JH, Lee YH. Hair greying is associated with active hair growth. The British Journal of Dermatology. 2011; 165: 1183–9.

19. Kelly CE, Thymiakou E, Dixon JE, Tanaka S, Godwin J, Episkopou V. Rnf165/Ark2C enhances BMP-Smad signaling to mediate motor axon extension. PLoS biology. 2013; 11: e1001538.

20. Shao W, Espenshade PJ. Expanding roles for SREBP in metabolism. Cell Metabolism. 2012; 16: 414–9.

21. Bou Sleiman M, Jha P, Houtkooper R, Williams RW, Wang X, Auwerx J. The Gene-Regulatory Footprint of Aging Highlights Conserved Central Regulators. Cell Reports. 2020; 32: 108203.

22. Zhang X, Zhang S, Liu X, Wang Y, Chang J, Zhang X, Mackintosh SG, Tackett AJ, He Y, Lv D, Laberge R-M, Campisi J, Wang J, et al. Oxidation resistance 1 is a novel senolytic target. Aging Cell. 2018; 17: e12780.

23. Cho SJ, Moon J-S, Lee C-M, Choi AMK, Stout-Delgado HW. Glucose Transporter 1-Dependent Glycolysis Is Increased during Aging-Related Lung Fibrosis, and Phloretin Inhibits Lung Fibrosis. American Journal of Respiratory Cell and Molecular Biology. 2017; 56: 521–31.

24. Park J-Y, Sohn H-Y, Koh YH, Jo C. Curcumin activates Nrf2 through PKCδ-mediated p62 phosphorylation at Ser351. Scientific Reports. 2021; 11: 8430.

25. Zhang L, Zhao J, Mu X, McGowan SJ, Angelini L, O'Kelly RD, Yousefzadeh MJ, Sakamoto A, Aversa Z, LeBrasseur NK, Suh Y, Huard J, Kamenecka TM, et al. Novel small

molecule inhibition of IKK/NF-κB activation reduces markers of senescence and improves healthspan in mouse models of aging. Aging Cell. 2021; 20: e13486.

26. Schaab EH, Crotti AEM, Iamamoto Y, Kato MJ, Lotufo LVC, Lopes NP. Biomimetic oxidation of piperine and piplartine catalyzed by iron(III) and manganese(III) porphyrins. Biol Pharm Bull. 2010; 33: 912–6.

27. Song M, Hwang JY, Lee MY, Jee J-G, Lee YM, Bae J-S, Kim JA, Lee SH, Lee S. In vitro inhibitory effect of piperlonguminine isolated from Piper longum on human cytochrome P450 1A2. Arch Pharm Res. 2014; 37: 1063–8.

28. Patel K, Chowdhury N, Doddapaneni R, Boakye CHA, Godugu C, Singh M. Piperlongumine for Enhancing Oral Bioavailability and Cytotoxicity of Docetaxel in Triple-Negative Breast Cancer. J Pharm Sci. 2015; 104: 4417–26.

29. Fofaria NM, Qhattal HSS, Liu X, Srivastava SK. Nanoemulsion formulations for anti-cancer agent piplartine--Characterization, toxicological, pharmacokinetics and efficacy studies. Int J Pharm. 2016; 498: 12–22.

30. Tripathi SK, Biswal BK. Piperlongumine, a potent anticancer phytotherapeutic: Perspectives on contemporary status and future possibilities as an anticancer agent. Pharmacol Res. 2020; 156: 104772.

31. Ghantous A, Sinjab A, Herceg Z, Darwiche N. Parthenolide: from plant shoots to cancer roots. Drug Discovery Today. 2013; 18: 894–905.

32. Guzman ML, Rossi RM, Neelakantan S, Li X, Corbett CA, Hassane DC, Becker MW, Bennett JM, Sullivan E, Lachowicz JL, Vaughan A, Sweeney CJ, Matthews W, et al. An orally bioavailable parthenolide analog selectively eradicates acute myelogenous leukemia stem and progenitor cells. Blood. 2007; 110: 4427–35.

33. Dei Cas M, Ghidoni R. Dietary Curcumin: Correlation between Bioavailability and Health Potential. Nutrients. 2019; 11: 2147.

34. Vareed SK, Kakarala M, Ruffin MT, Crowell JA, Normolle DP, Djuric Z, Brenner DE. Pharmacokinetics of curcumin conjugate metabolites in healthy human subjects. Cancer Epidemiol Biomarkers Prev. 2008; 17: 1411–7.

35. Jamwal R. Bioavailable curcumin formulations: A review of pharmacokinetic studies in healthy volunteers. J Integr Med. 2018; 16: 367–74.

36. Shoba G, Joy D, Joseph T, Majeed M, Rajendran R, Srinivas PS. Influence of piperine on the pharmacokinetics of curcumin in animals and human volunteers. Planta Med. 1998; 64: 353–6.

37. Zhao YY, Fan Y, Wang M, Wang J, Cheng JX, Zou JB, Zhang XF, Shi YJ, Guo DY. Studies on pharmacokinetic properties and absorption mechanism of phloretin: In vivo and in vitro. Biomed Pharmacother. 2020; 132: 110809.

38. Nakhate KT, Badwaik H, Choudhary R, Sakure K, Agrawal YO, Sharma C, Ojha S, Goyal SN. Therapeutic Potential and Pharmaceutical Development of a Multitargeted Flavonoid Phloretin. Nutrients. 2022; 14: 3638.

39. Wang Y, Li D, Lin H, Jiang S, Han L, Hou S, Lin S, Cheng Z, Bian W, Zhang X, He Y, Zhang K. Enhanced oral bioavailability and bioefficacy of phloretin using mixed polymeric modified self-nanoemulsions. Food Science & Nutrition. 2020; 8: 3545–58.
